# 2255. The Effect of Penicillin Allergy Labels on Perioperative Antibiotics for Elective Procedures

**DOI:** 10.1093/ofid/ofad500.1877

**Published:** 2023-11-27

**Authors:** Eileen Hasse, Minhua Zhang, Cosby A Stone, Sheena Marie Weaver, Jonathan Wanderer, Milner Staub, George E Nelson

**Affiliations:** Metro Infectious Disease Consultants, Chicago, Illinois; Vanderbilt University Medical Center, Brentwood, Tennessee; Vanderbilt University Medical Center, Brentwood, Tennessee; Vanderbilt University Medical Center, Brentwood, Tennessee; Vanderbilt University Medical Center, Brentwood, Tennessee; Vanderbilt University Medical Center, VA Tennessee Valley Healthcare System, Nashville, TN; Vanderbilt University Medical Center, Brentwood, Tennessee

## Abstract

**Background:**

As many as 20% of patients in the United States report a penicillin (PCN) allergy, of which 4% or less are true allergies. Previously, cephalosporins and PCN were estimated to have 10% cross-reactivity, but recent studies show lower rates. Patients with reported PCN allergy have increased surgical site and urinary tract infections, emergency room visits, and readmissions post-operatively, possibly due to unnecessary cefazolin avoidance. At Vanderbilt University Medical Center (VUMC), patients have an appointment with Vanderbilt Perioperative Evaluation Clinic (VPEC) to review medical history prior to elective procedures.

**Methods:**

In this retrospective cohort study, we evaluated patients ≥ 18 years old seen at VPEC prior to an elective procedure from 1/1/2022-1/1/2023. We excluded patients who did not receive perioperative antibiotics, did not undergo elective procedure, and patients with cephalosporin allergies. We compared demographics, clinical characteristics, and perioperative antibiotics received based on PCN allergy label presence in the electronic medical record (EMR). All variables were analyzed in R programming language with chi square or Fisher exact test.

**Figure 1.**
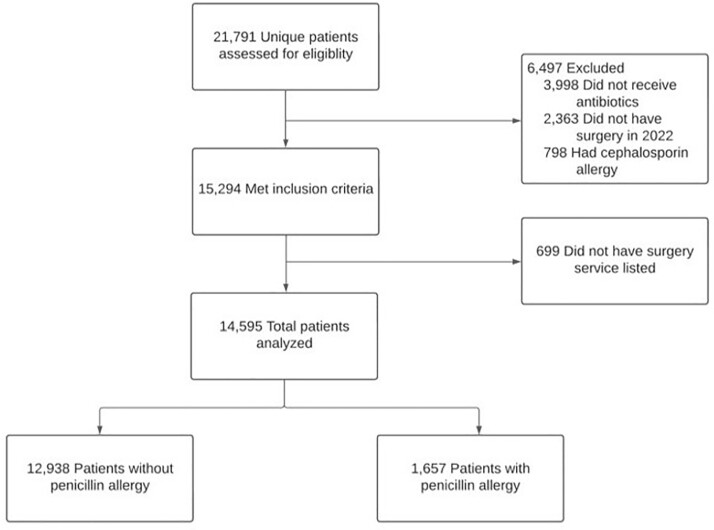
Exclusion Criteria

**Table 1.**
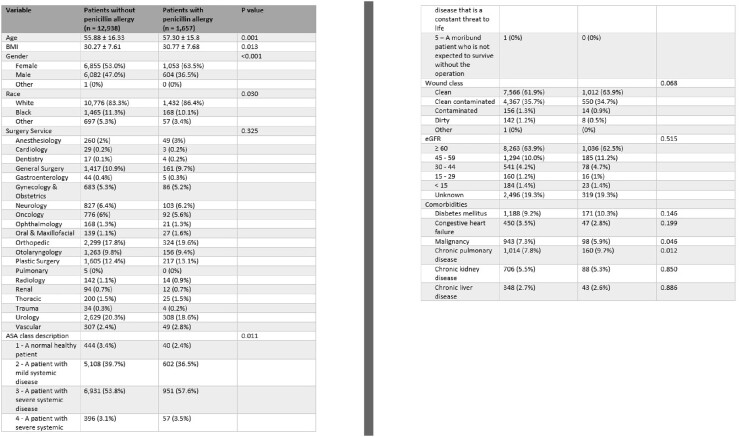
Demographics and Clinical Characteristics by Cohort

**Results:**

A total of 12,938 non-PCN allergy and 1,657 PCN allergy patients were included. Patients with PCN allergies were more often older (55 years vs 57 years; P = 0.001), women (53% vs 63.5%; P < 0.001), and white (83.3% vs 86.4%; P = 0.03). Antibiotic use differed between those with and without PCN allergies across all surgical services and in all classes except carbapenems, most notably cefazolin (80.4% vs 37.4%; P < 0.001), clindamycin (2.5% vs 48.3%; P < 0.001), and vancomycin (16.7% vs 19.5%; P = 0.005). There was also a difference in patients receiving ≥ 2 perioperative antibiotics (21% vs 32.5%; P < 0.001).

**Figure 2.**
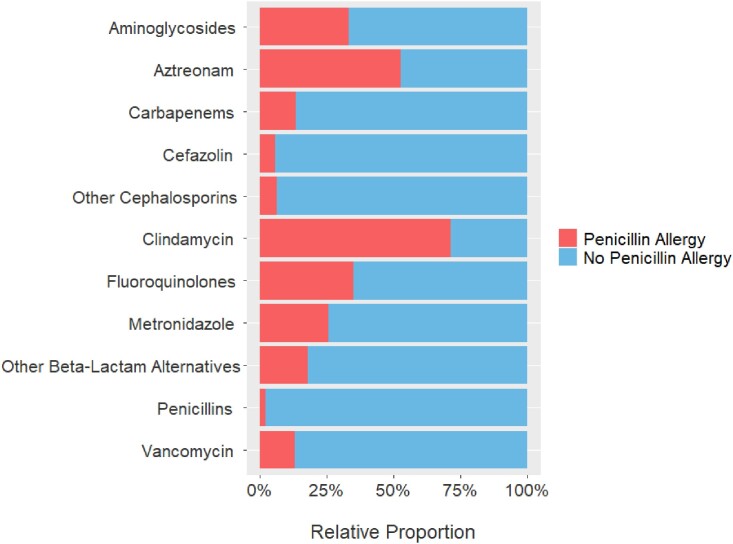
Relative Proportion of Antibiotic Administration by Cohort

**Figure 3.**
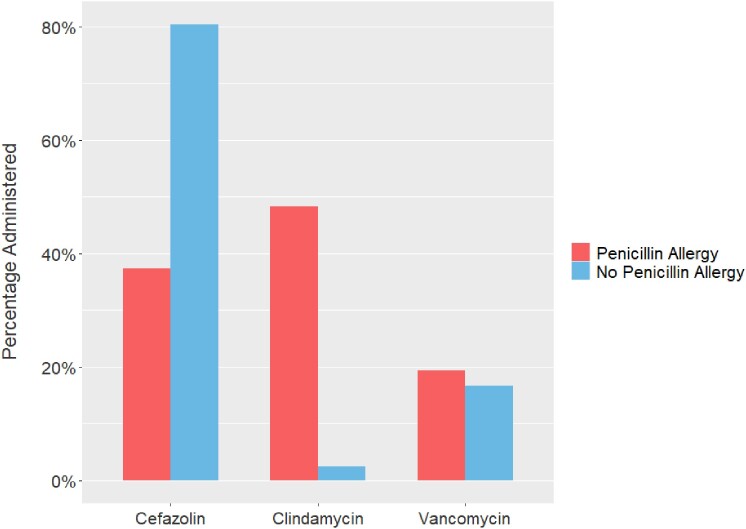
Cefazolin, Clindamycin, and Vancomycin Administration by Cohort

**Figure 4.**
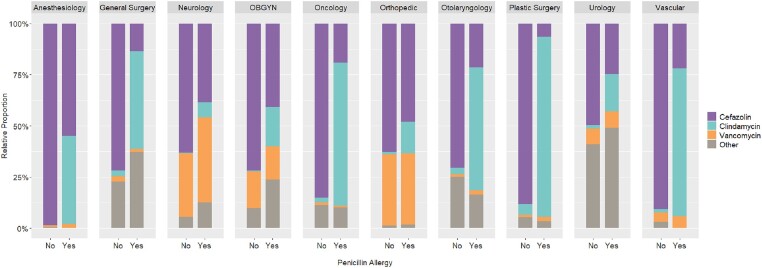
Relative Proportion of Cefazolin, Clindamycin, and Vancomycin Administration by Cohort and Surgical Service

**Conclusion:**

Patients with PCN allergies receive less perioperative cefazolin, more clindamycin, and more vancomycin for elective surgeries at VUMC. We have created a PCN allergy risk questionnaire to identify those with low-risk allergies at their VPEC appointment. This will be documented in their perioperative note and allergy section of the EMR. We plan to measure post-implementation cefazolin use, antibiotic exposure, and adverse outcomes.

**Disclosures:**

**Cosby A. Stone, Jr., MD, MPH**, American Academy of Allergy, Asthma, and Immunology Foundation: Grant/Research Support **Milner Staub, MD, MPH**, Gilead: Stocks/Bonds|Johnson & Johnson: Stocks/Bonds

